# Has the “green” transformation of the Common Agricultural Policy affected the environmental sustainability of farms?

**DOI:** 10.1371/journal.pone.0333703

**Published:** 2025-10-22

**Authors:** Aleksandra Pawłowska

**Affiliations:** Department of European Integration, Institute of Rural and Agricultural Development of the Polish Academy of Sciences, Warsaw, Poland; University of Salerno: Universita degli Studi di Salerno, ITALY

## Abstract

This study aims to quantify the level of environmental sustainability in EU farms using a synthetic measure of the environmental burden in agriculture and to assess the overall effectiveness of the “green” transformation of the Common Agricultural Policy (CAP) in enhancing farm sustainability. The Environmental Burden Index (EBI) was calculated for the average farm in a region using the TOPSIS method. The research hypothesis under investigation posits that environmental subsidies have contributed to a reduction in the environmental burden of agriculture. A panel regression model was estimated based on regional data from the EU FADN database covering the period 2004–2022 to identify the extent to which the “green” CAP transformation has impacted the environmental sustainability of farms. The research identified the EU regions that impose the lowest environmental burden in agriculture, with the leading regions primarily located in Italy. Furthermore, the estimated models revealed which types of subsidies benefit and hinder the environmental sustainability of farms. Notably, environmental subsidies were found to have a particularly positive impact on the EBI.

## 1. Introduction

The European Union’s Common Agricultural Policy (CAP), together with the Cohesion Policy, continues to represent a significant portion of the EU’s budget expenditures, accounting for about 30% [1]. Since the CAP was first introduced, there has been a continuous evolution in its priorities and instruments, reflecting a paradigm shift in agricultural and rural development from an industrial focus to a sustainable one. This shift moves away from viewing the agricultural sector as a closed system aimed solely at maximizing economic surplus from the farm’s perspective. Instead, it embraces a holistic approach that considers agricultural development as a living organism, integrating economic, social, and environmental objectives, along with intergenerational equity (cf. [2]). [3] identifies three categories of factors that shape the political discourse on agricultural and rural development. The first category consists of economic factors, such as income disparities between rural and urban populations, limited diversification into non-agricultural activities, and the unique economic characteristics of the agricultural sector. The second category includes social factors like the aging rural population, partial depopulation, high unemployment rates, and backwardness in various aspects of human activity. The third category comprises environmental factors, generally involving the negative impacts of agricultural activities and the pressing need for sustainable agricultural and rural development.

One of the most significant aspects of this evolution is the CAP’s “green” transformation, which has introduced a suite of environmental measures aimed at aligning agricultural practices with the EU’s sustainability and climate objectives. These include initiatives under the European Green Deal, such as the “Farm to Fork” strategy, which emphasizes sustainable food systems, and the integration of green payments tied to environmental and biodiversity conservation. Such measures represent an attempt to balance the demands of agricultural productivity with environmental protection and rural vitality. Considering the evolution of the CAP, it wonders to what extent the “green” transformation of this policy enhanced environmental sustainability by reducing the environmental burden in agriculture.

The purpose of this study is to quantify the average level of environmental sustainability of farms in the EU regions using the synthetic measure of environmental burden in agriculture and assess the overall effectiveness of “green” CAP transformation in increasing environmental sustainability of farms. Researchers acknowledge that environmental subsidies play a vital role in the sustainable advancement of agriculture. Therefore, the research hypothesis under investigation is that the environmental subsidies implemented thus far have contributed to a reduced environmental burden in agriculture. The novelty of this study lies in providing up-to-date and EU-wide quantitative analysis of the role of subsidies in reducing environmental burdens.

The paper is organized as follows. Section 1 outlines the conceptual foundations of farm sustainability, exploring the environmental, economic, and social dimensions, along with the synergies and trade-offs among them. Section 2 presents how CAP priorities and instruments evolved toward “green” transformation. Section 3 contains detailed information on the data utilized. Section 4 provides estimation results, whereas the discussion is encompassed within Section 5. The paper concludes with a summary that outlines the conclusions drawn from the analysis and its limitations.

## 2. Three dimensions of farm sustainability: Synergies and trade-offs

The idea of sustainability in agriculture arose from concerns about the long-term viability of farming practices and their environmental and social impacts. Although the term gained global prominence through the Brundtland Report [4], which defined sustainable development as “meeting the needs of the present without compromising the ability of future generations to meet their own needs”. Over time, particularly following the disruptive yet transformative Green Revolution, the sustainability discourse evolved from a narrow production-oriented focus to a multidimensional framework encompassing environmental, economic and social considerations [5].

Farm sustainability is typically analyzed within this framework through three distinct yet interrelated dimensions: environmental, economic and social [6,7]. Environmental sustainability pertains to the interaction of farms with natural ecosystems. It encompasses issues such as resource use efficiency, emission intensity, biodiversity conservation, and soil and water quality maintenance [8,9]. Indicators commonly used in this area include nutrient balance, greenhouse gas emissions, land-use change, and the adoption of agroecological practices [10,11]). Economic sustainability refers to a farm’s ability to remain financially viable and competitive. It is assessed through profitability, productivity, technical and allocative efficiency and the farm’s capacity to endure market fluctuations [12]. Social sustainability encompasses the well-being of individuals and communities involved in and affected by farm operations. It includes labor conditions, farmer and family welfare, generational renewal, social cohesion and involvement in local institutions [13].

Despite the frequent analytical separation of these three dimensions, numerous studies underscore their interdependence. Empirical evidence suggests that efforts to improve one dimension can reinforce or undermine the others, depending on the context. According to Herrera et al. [14], farms adopting technological innovations, integrated practices and advisory support are more likely to achieve simultaneous improvements in both environmental and economic performance. In Switzerland, diversified dairy-cattle systems achieved both productivity gains and reduced environmental impacts [15], while in Ireland, top-performing tillage and dairy farms combined high profitability with lower emission intensities [16,17]. Similarly, Polish farms undergoing economic growth have improved environmental performance [18]. Importantly, these synergies do not arise spontaneously but require institutional support, including access to information, favorable market conditions and tailored policy incentives. Farms that combine high investment capacity with knowledge and organizational flexibility are better positioned to implement practices that raise both productivity and environmental standards.

On the other hand, trade-offs are also prevalent. Intensification and specialization, often pursued to enhance economic returns, can negatively affect environmental or social outcomes. Studies from Spain and Poland indicate that economic efficiency gains may come due to environmental degradation or reduced social cohesion [19,20]. Conversely, while organic farming often scores highly on environmental indicators such as biodiversity preservation and reduced chemical use, its lower yields and higher labor intensity can result in reduced economic returns and limited scalability [21]. Moreover, large-scale specialized farms in the Netherlands demonstrated improved economic and environmental performance but faced declines in social indicators such as worker satisfaction and community engagement [11]. In some contexts, public support schemes mitigate these drawbacks. Nevertheless, their long-term effectiveness remains debated, especially in competitive market environments.

## 3. “Green” transformation of the CAP

The idea of sustainability has shaped the reform path of the CAP, leading to a broader redefinition of its aims beyond just economic objectives. For many years, the political discourse surrounding the CAP was dominated by productivism, which viewed agriculture’s primary role as food production [22]. Under this paradigm, the CAP aimed to stimulate agricultural output and increase sector productivity. Rooted in neo-mercantilist tradition, early CAP policies included import protection and export subsidies to support agricultural prices. The agricultural lobby justified public funds being transferred to farmers to ensure food security, improve productivity, and raise farm incomes, advocating for protectionism and state support for agriculture as a strategic sector [23,24]. Consequently, during the 1970s and 1980s, the CAP became an instrument of sectoral protectionism, leading to negative outcomes like overproduction and environmental degradation due to intensive farming practices encouraged by high price supports [25]. The first significant reform was the Mansholt Plan, which sought to increase the size of farms and reduce food surpluses (see: [26]). This reform aimed to improve agrarian structures by favoring farms with substantial production potential and eliminating inefficient ones.

In 1992, the MacSharry Plan addressed issues such as overproduction, low farm incomes, environmental damage, and escalating agricultural budget expenditures [27]. The reform proposed a model of competitive and efficient agriculture based on family farms engaged in both production and environmental conservation. It involved reducing protectionist policies, exposing agriculture to market, and decoupling subsidies from production by introducing direct payments to compensate for income losses due to lowered guaranteed prices.

Amid implementing the MacSharry Reforms, the 1996 Cork Declaration emphasized the need for rural development through ten principles collectively known as “A Living Countryside” [28]. The declaration called for prioritizing rural development to enhance the well-being of rural populations, adopting an integrated rural policy approach, promoting diverse economic and social activities, conserving natural resources and cultural identity, respecting subsidiarity, simplifying related legislation, integrating measures into single regional development programs, leveraging local financial resources, enhancing administrative capacities of local and regional authorities, and ensuring proper use of public funds through monitoring, research, innovation, and public engagement.

Criticism of productivism led to multifunctionality dominating European political discourse, culminating in the adoption of the European Model of Agriculture by most MSs in 1997 [29]. This model argues that agriculture requires special treatment because it provides public goods such as maintaining rural landscapes, preserving cultural heritage, and protecting the environment and biodiversity (cf. [30]). Since public goods are inadequately regulated by market mechanisms, state intervention is necessary to correct negative externalities and encourage farmers to supply these goods [31]. Following this shift, the Agenda 2000 reform addressed challenges like trade liberalization in agri-food products, rising global demand, environmental concerns, food security, and the impending enlargement of the EU [32]. The Fischler Reform (Luxembourg Reform) of 2003 revisited direct subsidies, advocating for their decoupling from production. The goal was to strengthen rural development by introducing a single flat-rate direct payment conditional on compliance with environmental, animal welfare, and food safety standards – a principle known as cross-compliance. Production-linked payments were abolished and integrated into the single payment scheme, and market interventions were relaxed to allow farms to respond to market signals [33]. EU agriculture began to move towards post-productivism, emphasizing sustainable practices that are economically and socially connected to rural communities rather than focusing on intensive production [34]. As a result, the CAP increasingly incorporated “green” payments to support this new direction.

In the late 20th century, a resurgence of productivism emerged in the form of neo-productivism, currently present in the EU political discourse. [35] attribute this shift to factors such as the 2007–2008 food crisis, the diversion of food production to non-agricultural purposes (e.g., biofuels), changing consumer preferences, food waste, climate change, diminishing access to water resources, soil degradation, loss of biodiversity, and transformations in global food systems. According to [36,37], neo-productivism is characterized by reducing state intervention in markets while ideologically promoting environmentally friendly agriculture, decreasing agricultural intensification in favor of more sustainable practices, diversifying income sources, and emphasizing environmental protection. Grochowska [38] observes that neo-productivism is evident in EU political discourse through references to sustainability principles. However, the promotion of “sustainable agricultural intensification” in this context is seen as somewhat paradoxical. This concept focuses on increasing agricultural production using existing land with minimal environmental impact. While well-intentioned, it has been employed by the agricultural lobby to continue the previous model of production intensification, merely rebranded with different terminology.

Significant changes to the CAP were introduced in the 2014–2020 financial period, emphasizing three main objectives: increasing agricultural productivity and competitiveness, sustainable management of natural resources and climate action, and balanced territorial development of rural communities and economies. A key aspect of this reform was transforming subsidies unrelated to production into a multifunctional support system. An example is the “greening” of the CAP, which integrated environmental measures more deeply into the direct payment system. In 2016, the second Cork Declaration, titled “A Better Life in Rural Areas”, was adopted [39]. It outlined directions for rural development and agricultural policy, including promoting rural prosperity, strengthening value chains, investing in viability and vitality, protecting the rural environment, sustainable management of natural resources, supporting climate action, enhancing knowledge and innovation, improving rural governance, simplifying policy implementation, and increasing effectiveness and accountability.

The CAP 2023–2027, implemented through Strategic Plans, represents a significant policy shift, aiming to support a smart and resilient agricultural sector through research and innovation, greater concern for the climate and environment, and strengthening the socio-economic structure of rural areas. The outcomes of these Strategic Plans aim to contribute to the EU’s environmental and climate commitments and align with objectives outlined in the European Green Deal [40], the “Farm to Fork” strategy [41], and the Biodiversity Strategy for 2030 [42].

## 4. Materials and methods

### 4.1. Data

The study used the region-average farm data from the EU FADN (Farm Accountancy Data Network) for the years 2004–2022. The panel data was unbalanced because there was no data available for Germany, Greece, Spain, Malta, Slovenia, and Croatia in 2022. The environmental sustainability of the region was assessed using the Environmental Burden Index (EBI), calculated according to the methodology provided by Czyżewski, M atuszczak and Muntean [43]. Table 1 presents the variables from the EU FADN database applied for the construction of the EBI at the level of an average farm in a given region (EU FADN codes in brackets).

**Table 1:** Descriptive statistics of the selected components of the Environmental Burden Index (source: [44]).

Variable	Definition
Stock density per ha (SE120 [LU/ha])	Density of ruminant grazing livestock: average number of LU of bovines, sheep and goats per hectare of forage UAA. Forage area includes fodder crops, agricultural fallows and land withdrawn from production (not cultivated, except in the exceptional cases of crops under set- aside schemes). Stocking density is calculated only for holdings with corresponding animals and with forage area.
Mineral fertilizers use per ha (SE295 [EUR/farm]/SE025 [ha/farm])	Purchased fertilisers and soil improvers (excluding those used for forests).
Plant protection products use per ha (SE300 [EUR/farm]/SE025 [ha/farm])	Plant protection products, traps and baits, bird scarers, anti-hail shells, frost protection, etc. (excluding those used for forests).
Total use of energy per ha (SE345 [EUR/farm]/SE025 [ha/farm])	Motor fuels and lubricants, electricity, heating fuels.
Woodland area in relation to utilized agricultural area (UAA) (SE075 [ha/farm]/SE025 [ha/farm])	Woodland area, forests, poplar plantations, including nurseries. Not included in UAA (SE025).

All variables were converted into destimulants of the environmental quality, except the last one.

According to Czyżewski, Matuszczak and Muntean [43], operationalizing the environmental burden in agriculture is a problematic issue. Reytar, Hanson, and Henninger [45] identified indicators of sustainable agriculture based on two dimensions: the stages of the “causal chain” that these indicators can influence (including public policy, farmer practices, and biophysical performance) and thematic areas such as water, climate change, land conversion (terrestrial ecosystems), pollution (nutrients, pesticides), and soil health. Czyżewski, Matuszczak and Muntean [43] contended that the chosen indicators from the EU FADN database correspond with the discussion on agricultural environmental sustainability presented by Latruffe et al. [12].

The EBI was calculated for an average farm in a region using the TOPSIS (Technique for Order of Preference by Similarity to Ideal Solution) method. The construction of Hwang and Yoon’s TOPSIS synthetic measure is as follows [46]:


$${\text{q}}_{\text{i}}=\frac{\sqrt{\sum_{\text{j}=\text{1}}^{\text{m}}{\left({\text{z}}_{\text{ij}}-{\text{z}}_{\text{0j}}^{-}\right)}^{\text{2}}}}{\sqrt{\sum_{\text{j}=\text{1}}^{\text{m}}{\left({\text{z}}_{\text{ij}}-{\text{z}}_{\text{0j}}^{+}\right)}^{\text{2}}}+\sqrt{\sum_{\text{j}=\text{1}}^{\text{m}}{\left({\text{z}}_{\text{ij}}-{\text{z}}_{\text{0j}}^{-}\right)}^{\text{2}}}}$$ (1)

where:


$$\text{positive}\;\text{ideal}\;\text{solution}\;\;:\;{\text{z}}_{\text{0j}}^{+}=\left\{ \begin{array}{c} {\text{max}}_{\text{i}}\left\{{\text{z}}_{\text{ij}}\right\}\;\text{for}\;\text{stimulants}\\ {\text{min}}_{\text{i}}\left\{{\text{z}}_{\text{ij}}\right\}\;\text{for}\;\text{de}\text{stimulants} \end{array}\right.\;$$ (2)

$$\text{negative}\;\text{ideal}\;\text{solution}\;\;:\;{\text{z}}_{\text{0j}}^{-}=\left\{ \begin{array}{c} {\text{min}}_{\text{i}}\left\{{\text{z}}_{\text{ij}}\right\}\;\text{for}\;\text{stimulants}\\ {\text{max}}_{\text{i}}\left\{{\text{z}}_{\text{ij}}\right\}\;\text{for}\;\text{de}\text{stimulants} \end{array}\right.\;$$ (3)

$$\text{normalized}\;\text{variable}\;\;:\;{\text{z}}_{\text{ij}}=\frac{{\text{x}}_{\text{ij}}}{\sqrt{\sum_{\text{i}=\text{1}}^{\text{n}}{\text{x}}_{\text{ij}}^{\text{2}}}}$$ (4)

and:

${\text{x}}_{\text{ij}}$ – value of the j*th* variable for the i*th* observation (region) and ${\text{q}}_{\text{i}}\in [\text{0};\text{1}].$ The higher the value of the index, the lower the pressure on the environment.

Table 2 presents descriptive statistics of the components of the EBI.

**Table 2:** Descriptive statistics of the selected components of the Environmental Burden Index (source: own calculation).

Variable	Mean		Median		Standard deviation		Min		Max	
	2004	2022	2004	2022	2004	2022	2004	2022	2004	2022
SE120	1.81	1.33	1.32	1.19	2.01	0.70	0.27	0.32	13.04	4.38
SE295/SE025	104.21	206.85	83.56	180.15	137.21	133.02	3.44	5.30	1,215.92	842.48
SE300/SE025	85.50	102.22	62.09	79.81	120.92	77.03	1.26	5.51	1,139.18	437.21
SE345/SE025	149.27	247.44	103.78	207.52	282.99	175.42	12.20	60.19	2,976.53	1,325.71
SE075/SE025	0.07	0.05	0.01	0.00	0.17	0.17	0.00	0.00	1.18	1.44

In EU regions, the only notable decrease during the analyzed period was in stock density. In 2004, the average stock density stood at 1.81 LU/ha, which fell to 1.33 LU/ha by 2022, potentially suggesting a shift towards less intensive animal farming practices. Moreover, the proportion of woodland area to UAA slightly diminished from 0.07 to 0.05 over the analyzed period. Simultaneously, the average use of mineral fertilizers per hectare nearly doubled, rising from 104.21 EUR/ha to 206.85 EUR/ha. The average usage of plant protection products also increased from 85.50 EUR/ha to 102.22 EUR/ha between 2004 and 2022. Furthermore, energy consumption per hectare escalated from 149.27 EUR/ha in 2004 to 247.44 EUR/ha in 2022.

### 4.2. Variables

**Stock density.** The selection of an appropriate stocking density is essential for preserving soil quality and ensuring the long-term sustainability of grassland systems [47]. Grazing management is essential for ecological sustainability and environmental quality (especially after long-term grazing), affecting key soil properties, including pH levels, soil organic matter and soil nutrient concentrations [48,49]. The effect of grazing on soil quality relies heavily on choosing the right stocking density [50,51]. When stocking density surpasses the “carrying capacity”, it can negatively affect the long-term sustainability of a grassland system [52].

**Mineral fertilizers.** Studies have shown that agriculture contributes approximately 10–12% of global greenhouse gas emissions, with about 38% of these emissions resulting from the application of organic or mineral fertilizers [53–55]. Additionally, fertilizers are a primary source of nutrient pollution, leading to eutrophication in water bodies, which causes algal blooms and subsequent oxygen depletion, adversely affecting aquatic ecosystems [56].

**Plant protection.** Plant protection includes, among others, plant production products (PPPs). PPP can significantly harm the environment, especially biodiversity [57]. In the EU, no PPP can be used unless it has first been scientifically established that (1) they have no harmful effects on consumers, farmers and local residents and passers-by; (2) they do not cause unacceptable effects on the environment; (3) they are sufficiently effective against pests [58].

**Use of energy.** Modern farming relies heavily on fossil fuels for machinery operation and the production of inputs like fertilizers and pesticides, leading to substantial greenhouse gas emissions [59].

**Woodland area.** Woodlands contribute to carbon sequestration, thereby mitigating climate change by absorbing carbon dioxide from the atmosphere. Additionally, integrating trees into agricultural landscapes through practices like agroforestry enhances biodiversity, improves soil structure, and reduces erosion. Furthermore, silvopasture systems, which combine forestry and grazing, can increase overall productivity and provide environmental benefits [60].

### 4.3. Model specification

In the final step of the analysis, a panel regression model was estimated, based on 2476 observations, encompassing EU FADN regions over the period 2004–2022, to identify to what extent “green” CAP payments impacted the environmental burden in agriculture. The paper employed regression with time fixed effects, controlling for subsidies that are constant across farms but vary over time. This assumption stems from the study’s goal to evaluate the “green” transformation of the CAP. The general form of a model is presented in Eq (5) following [61].


$$\begin{array}{c} Y_{it}=\beta_{\text{0}}+\sum {\mathrm{\backslash nolimits}}_{k=\text{1}}^{K}\beta_{k}X_{k,it}+\sum {\mathrm{\backslash nolimits}}_{t=\text{2}}^{T}\delta_{t}D_{t}+u_{it} \end{array}$$ (5)

Where: Y – output, X – vector of continuous variables capturing CAP payments, Z – vector of T-1 dummy variables and where $D_{\text{2}}=\text{1}$ if t = 2 and $D_{\text{2}}=\text{0}$ otherwise, and so forth.

The model is specified as follows:


$$\begin{array}{c} {EBI}_{it}=\beta_{\text{0}}+\beta_{\text{1}}{SE\text{4}\text{0}\text{6}}_{it}+\beta_{\text{2}}{SE\text{6}\text{2}\text{1}}_{it}+\beta_{\text{3}}{SE\text{6}\text{2}\text{2}}_{it}+\beta_{\text{4}}{SE\text{6}\text{2}\text{3}}_{it}+\beta_{\text{5}}{SE\text{5}\text{2}\text{5}}_{it}+\beta_{\text{6}}{SE\text{6}\text{2}\text{6}}_{it}+\sum {\mathrm{\backslash nolimits}}_{t=\text{2}}^{T}\delta_{l}D_{t}+u_{it} \end{array}$$ (6)

Where:

EBI – Environmental Burden Index calculating according Eq (1),

SE406 – subsidies on investments [EUR] – the amount of subsidies on investments allocated to the accounting year,

SE621 – environmental subsidies [EUR] – subsidies on environment and environmental restrictions,

SE622 – Less Favored Area (LFA) subsidies [EUR] – subsidies for LFA/areas facing natural or other specific constraints (such as areas with natural constraints),

SE623 – other rural development payments [EUR] – support to help farmers to adapt to standards, to use farm advisory services, to improve the quality of agricultural products, training, afforestation and ecological stability of forests,

SE625 – subsidies on intermediate consumption [EUR] – all farm subsidies on intermediate consumption,

SE626 – subsidies on external factors [EUR] – subsidies on wages, rent and interests.

Subsidies with a Variance Inflation Factor (VIF) exceeding five were not included in the analysis. This exclusion applied to direct payments (SE606), subsidies on crops (SE610), subsidies on livestock (SE615), decoupled payments (SE630), and other subsidies (SE699). The model was estimated using the fixed-effects panel regression method, which gives the ability to control for individual specific unobservable characteristics. The choice between the panel regression model and the Ordinary Least Squares regression was made using the Breusch-Pagan test, which assessed the necessity of accounting for panel-specific variations [62]. Furthermore, the Hausman test was employed to determine the appropriate specification between fixed-effects and random-effects models [63]. The use of fixed-effects panel regression also corresponds to the stated goal, i.e., to analyze the “green” transformation of the CAP.

### 4.4. Software

The study was conducted using R [64] and packages plm [65], regclass [66] and lmtest [67].

## 5. Results

Following the analysis, EU regions were ranked based on a composite measure of environmental burden, assessed for the average farm in the EUFADN region. Table 3 shows that five of the “Top 10” regions from 2004 reappeared in 2022. It concerned four regions from Italy (Valle d’Aosta, Toscana, Umbria and Alto Adige) and Austria. The 2022 “Top 10” list shows greater diversity, featuring regions from France, Estonia, and Latvia – countries that were not represented in the 2004 list.

**Table 3:** Top ten and bottom ten environmentally sustainable EUFADN regions according to the Environmental Burden Index (source: own calculation).

2004			2022	
Region		Score	Region	Score
Top 10	Slovenia (SI)	0.930	Alto Adige (IT)	0.807
	Alto Adige (IT)	0.892	Umbria (IT)	0.537
	Közép-Dunántúl (HU)	0.799	Austria (AT)	0.526
	Austria (AT)	0.785	Toscana (IT)	0.508
	Sicilia (IT)	0.780	Valle d’Aosta (IT)	0.460
	Ribatejo e Oeste (PT)	0.773	Alentejo e Algarve (PT)	0.458
	Umbria (IT)	0.765	Sardegna (IT)	0.453
	Entre Douro e Minho (PT)	0.763	Latvia (LV)	0.448
	Toscana (IT)	0.757	Corse (FR)	0.446
	Valle d’Aosta (IT)	0.743	Estonia (EE)	0.445
Bottom 10	Liguria (IT)	0.662	Cyprus (CY)	0.369
	Vlaanderen (BE)	0.655	Nord-Pas-de-Calais (FR)	0.357
	Castilla-La Mancha (ES)	0.642	Trentino (IT)	0.331
	Thessalia (EL)	0.640	Lombardia (IT)	0.322
	The Netherlands (NL)	0.639	Veneto (IT)	0.306
	Sterea Ellas-Nissi Egaeou-Kriti (EL)	0.634	Guadeloupe (FR)	0.288
	Makedonia-Thraki (EL)	0.617	Vlaanderen (BE)	0.287
	Malta (MT)	0.562	La Réunion (FR)	0.278
	Canarias (ES)	0.425	Liguria (IT)	0.266
	Hamburg (DE)	0.312	The Netherlands (NL)	0.217

**Fig 1:**
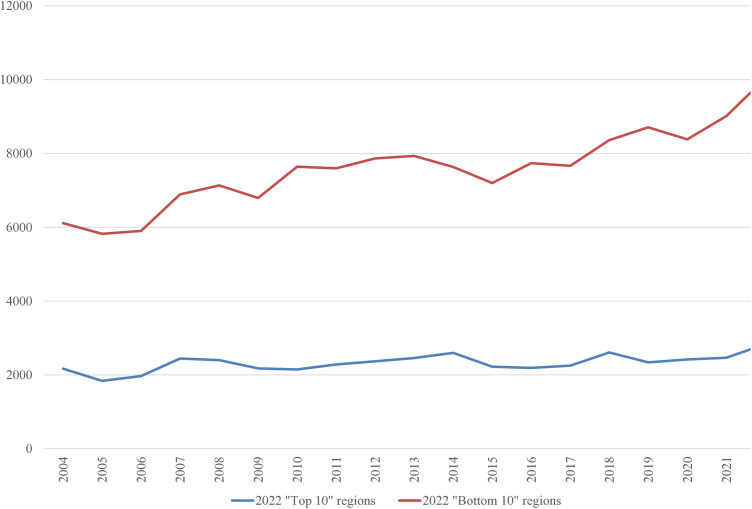
Average production per Utilized Agricultural Area in 2004-2022 (EUR/ha).

**Fig 2:**
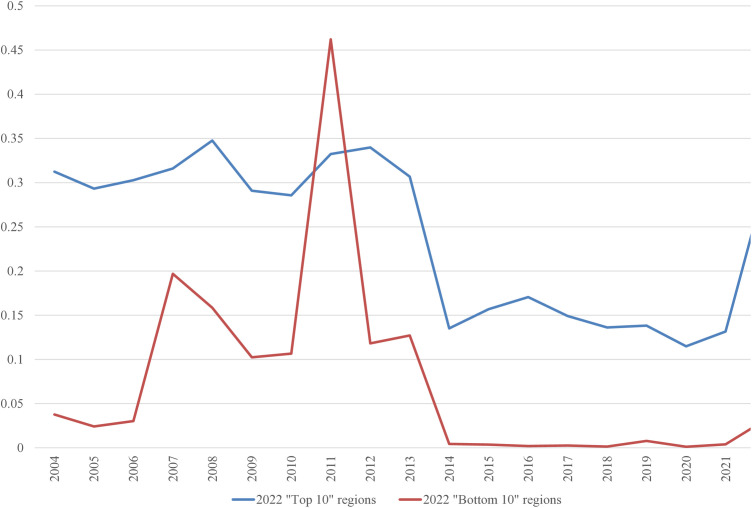
Average woodland area per Utilized Agricultural Area in 2004-2022 (ha/ha).

**Fig 3:**
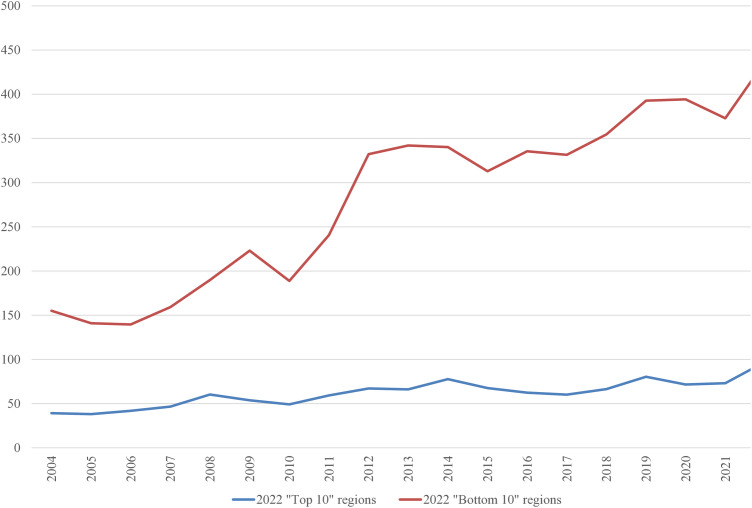
Average fertilizers use per Utilized Agricultural Area in 2004-2022 (EUR/ha).

**Fig 4:**
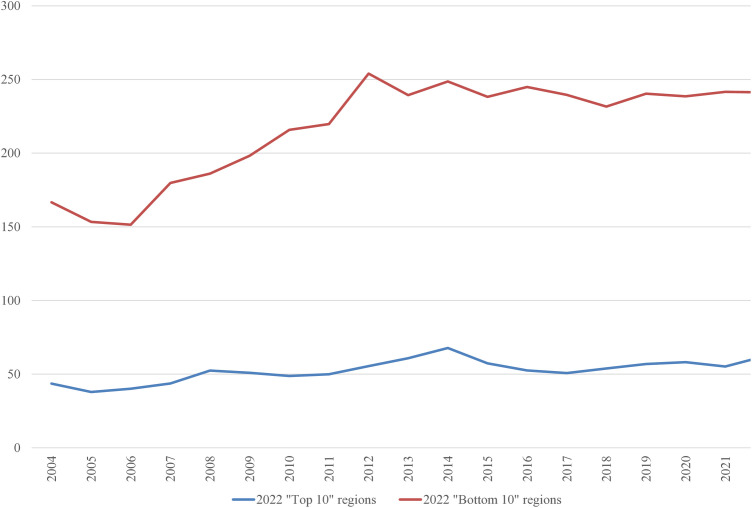
Average plant protection products per Utilized Agricultural Area in 2004-2022 (EUR/ha).

**Fig 5:**
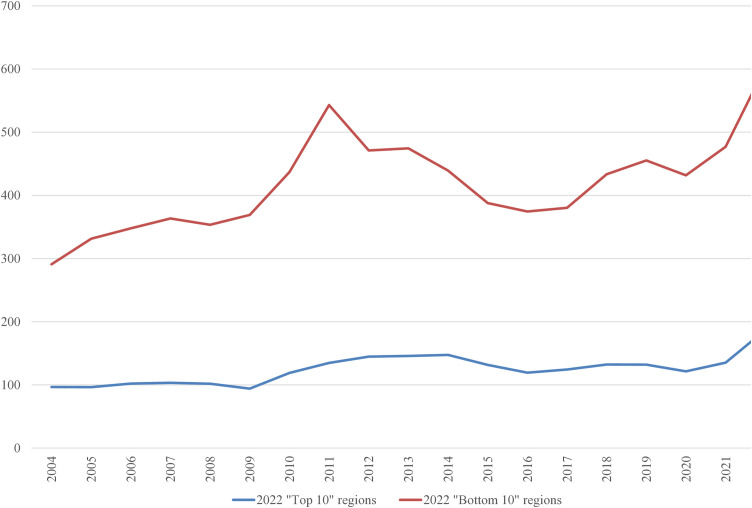
Average energy use per Utilized Agricultural Area in 2004-2022 (EUR/ha).

**Fig 6:**
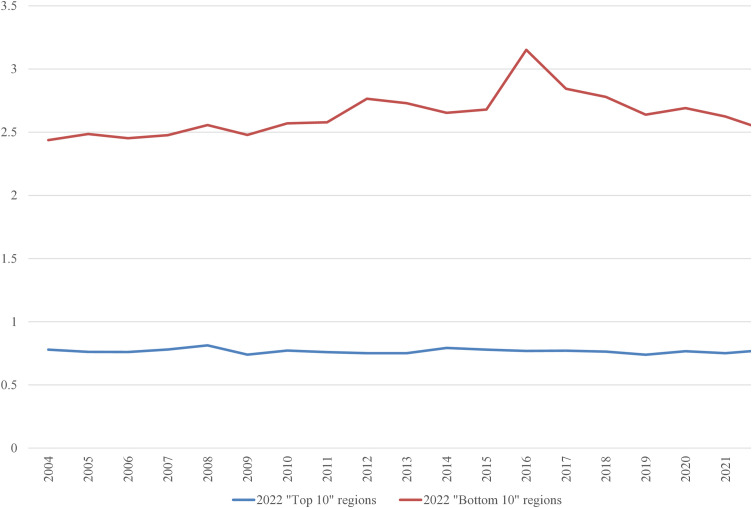
Average stock density in 2004-2022 (LU/ha).

**Fig 7:**
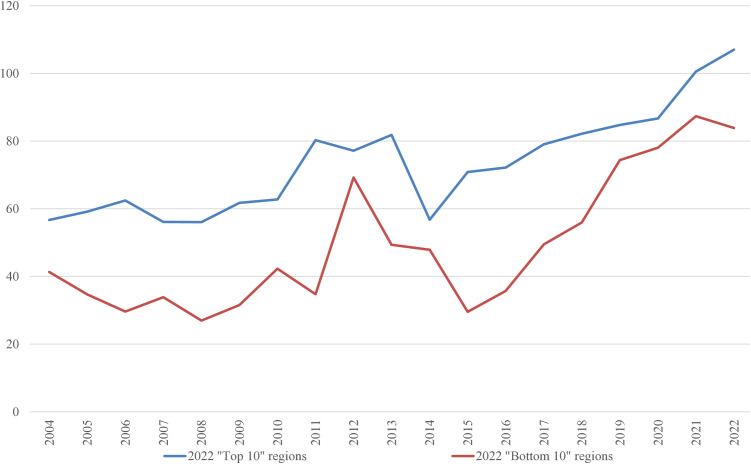
Average environmental subsidies under CAP per Utilized Agricultural Area in 2004-2022 (EUR/ha).

**Fig 8:**
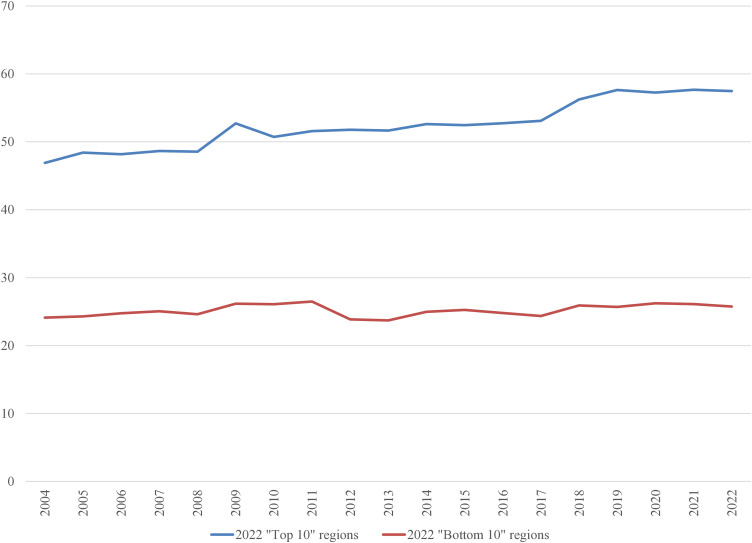
Average Utilized Agricultural Area in 2004-2022 (ha).

In the 2022 “Bottom 10” list, only three regions were repeated from the 2004 list: the Netherlands, Liguria (Italy), and Vlaanderen (Belgium). It is worth examining the average inputs that contribute to the ranking. These are depicted in Figs 1–8, standardized per hectare. Alongside the ranking components, the total amount of rural development subsidies is also included due to the focus of the analysis. This category encompasses environmental subsidies, LFA/ANC subsidies, and other rural development payments, which include national support for rural development.

A notable disparity existed between the “Top 10” and “Bottom 10” regions in terms of production intensity. The farms creating the lowest environmental burden tended to be extensive in their production practices, while those identified as least environmentally sustainable exhibited, on average, three times higher production per hectare. At the same time, the average UAA was three times higher in the “Top 10” farms compared to the “Bottom 10” farms. This surprising finding contradicts the prevailing belief that smaller farms are environmentally sustainable (cf. [43]). The disparities between the two groups concerning woodland, energy, fertilizers, plant protection products, and stock density are no longer surprising. Except for the first variable, which is a stimulant, the other variables tended to have higher average values in the “Bottom 10” group. In addition to the variables used to determine the EIB, differences in environmental subsidies per hectare were also looked at. If one assumes (after [43]) that their amount is indicative of payments for the provision of public goods, then the differences between the “Top 10” and “Bottom 10” groups are not significant, especially in the last three years.

Hence, the final stage of the analysis examined whether there was a relationship between the different types of subsidies per farm (Table 4) and per hectare (Table 5) and the level of environmental sustainability of farms, calculated using EBI. The purpose of this analysis was not to identify the determinants of EBI variability but only to test the direction and strength of the (linear) relationship between this index and CAP payments, with particular emphasis on environmental subsidies. For both models, the R-square was about 12%, which is a relatively good fit, given that agricultural policy is only one of the factors affecting environmental sustainability. The robustness check involved estimating models for the balanced panel from 2004 to 2021, leading to consistent conclusions.

**Table 4:** Results of the panel regression model with fixed effects (n = 2476) (source: own calculation).

Coefficient	Estimate	Standard Error	t	p-value
SE406	−0.000000007	0.000000629	−0.0116	0.990750
SE621	0.000001553	0.000000476	3.2614	0.001124
SE622	−0.000000009	0.000000394	−0.0248	0.980212
SE623	−0.000001286	0.000002478	−0.5187	0.603995
SE625	−0.000000513	0.000000711	−0.7223	0.470203
SE626	−0.000006833	0.000002792	−2.4472	0.014466

**Table 5:** Results of the panel regression model with fixed effects (n = 2476) (source: own calculation).

Coefficient	Estimate	Standard Error	t	p-value
SE406/ha	−0.000055208	0.000017751	−3.1101	0.001891
SE621/ha	0.000259622	0.000030428	8.5323	<0.0001
SE622/ha	0.000004106	0.000025580	0.1605	0.872478
SE623/ha	−0.000265986	0.000029768	−8.9352	<0.0001
SE625/ha	0.000135608	0.000050069	2.7084	0.006807
SE626/ha	−0.002246524	0.000167606	−13.4036	<0.0001

The estimated models indicate the types of payments that support the environmental sustainability of farms. A significant relationship (assuming a significance level of 0.05) between the EBI and CAP payments was noted for environmental subsidies and subsidies on external factors (wages, rent and interests). In the former case, the effect was positive – that is, an increase in environmental subsidies led to a rise in the EBI, meaning creating a lower environmental burden in agriculture. Conversely, the latter had a negative impact. However, this conclusion was drawn from calculations based on the absolute values of the subsidies. When examining payments per hectare, a positive impact on the EBI was observed for both environmental subsidies and subsidies related to intermediate consumption. The negative impact was observed, in turn, from subsidies that generally lead to an increase in productivity and capital-intensive production techniques, such as subsidies on investments and subsidies on external factors. What is surprising here is the negative impact of other subsidies for rural development, which are dedicated to afforestation and the ecological stability of forests.

## 6. Discussion

The results mainly support the conclusions drawn by Czyżewski,Matuszczak, and Guth [68], as the authors examined the effect of various types of CAP payments on the Environmental Burden Index. Furthermore, this study also aligns with other research on how agricultural policy affects environmental farm sustainability, even though the methods for its operationalization vary. This finding reflects a broader range of evidence indicating that when payments support public-good provision, farms can enhance eco-efficiency while maintaining their income [69]. According to Bernini and Galli [70], based on data on Italian farms, CAP subsidies boost farms’ environmental sustainability at the expense of economic inefficiency. Rudnicki et al. [71], using Poland as an example, noted that CAP subsidies significantly influence the environmental sustainability of farms by promoting pro-environmental practices, such as organic farming and afforestation. Additionally, the research supports the conclusions drawn by Bonfiglio, Arzeni, and Bodini [72], regarding the role of agri-environmental schemes in enhancing eco-efficiency on farms. Czyżewski et al. [73] reached similar conclusions regarding the positive (cross-sectional) impact of these payments on the environmental sustainable value, while also highlighting the beneficial effects of investment subsidies on this value. Simultaneously, the limited explanatory power of the regressions aligns with earlier studies indicating that policy is just one of several factors influencing sustainability outcomes, alongside natural conditions, market integration, and knowledge availability [74].

However, certain methodological limitations should be acknowledged. Firstly, a limitation pertains to potential selection bias arising from the use of aggregated data at the regional level. Although using averaged EU FADN data ensures comparability across the EU, it may obscure variability among farms within a given region. Regions often exhibit heterogeneity in terms of farm structure, production types or environmental practices. Consequently, relying on regional averages may lead to oversimplified conclusions regarding the actual environmental burden at the individual farm level. Secondly, attention should be given to the sensitivity of the EBI to variations across agricultural sectors. Variables employed in the analysis, such as stocking density, fertilizer use and plant protection products, might have different implications depending on the agricultural sector. For instance, livestock-oriented farms naturally exhibit higher stocking density, whereas crop-oriented farms might present elevated levels of plant protection products. Therefore, inter-regional comparisons may be skewed due to variations in agricultural production structures.

## 7. Conclusions

The research conducted identified the EU regions that impose the lowest environmental burden in agriculture, with the leading regions primarily located in Italy. This is particularly attributed to the level of forest cover in these areas, which plays a crucial role in enhancing environmental sustainability. The estimated models revealed that various types of subsidies can either benefit or hinder the environmental sustainability of farms. Notably, environmental subsidies had a particularly positive impact on the EBI, confirming the initial research hypothesis and supporting the conclusions of various studies on the relationship between CAP payments and environmental sustainability.

The introduction of pro-environmental measures within the CAP was intended to benefit the environment, but this was not the policy’s primary goal. For instance, the set-aside program, launched in 1992 to address agricultural overproduction, was gradually phased out as market conditions shifted, reaching zero in 2008 and eventually being eliminated in 2013. Meanwhile, agri-environmental programs, also introduced in 1992 and specifically aimed at mitigating agriculture’s environmental impact, were optional for farmers and regarded as supplementary initiatives. Although environmental concerns became more prominent in political discourse, implementing effective measures proved challenging due to the low prioritization of environmental protection and the dominance of a closed agricultural policy network that primarily served the interests of agricultural producers [75].The findings underscore the necessity for a shift in policy focus toward prioritizing eco-friendly practices, which are increasingly recognized as vital for the sustainable transformation of agriculture. The historical context reveals a struggle between economic interests and genuine environmental protection. Moving forward, it is essential to prioritize policies that promote eco-friendly practices. Considering this, the CAP 2023–2027 introduced the Green Architecture, which integrates mandatory conditionality requirements with voluntary interventions, including Eco-schemes. Evidence from countries like Austria, Ireland, and the Netherlands highlights that flexible, locally adaptable approaches and collective actions significantly enhance farmer participation and policy effectiveness [76]. Future studies should concentrate on assessing the lasting effectiveness of these Green Architecture strategies, particularly their actual environmental effects, and improving policies informed by new evidence.

## Supporting information

S1 File PLOS_EUFADN_region. (XLSX)
